# A map of words: Retrieving the spatial layout of medium-scale geographical maps through distributional semantics

**DOI:** 10.1016/j.neuropsychologia.2025.109190

**Published:** 2025-06-03

**Authors:** Giorgia Anceresi, Daniele Gatti, Tomaso Vecchi, Marco Marelli, Luca Rinaldi

**Affiliations:** a Department of Brain and Behavioral Sciences, University of Pavia, Piazza Botta 6, 27100, Pavia, Italy; b Cognitive Psychology Unit, IRCCS Mondino Foundation, via Mondino 2, 27100, Pavia, Italy; c Department of Psychology, University of Milano-Bicocca, Piazza dell’Ateneo Nuovo 1, 20126, Milano, Italy; d NeuroMI, Milan Center for Neuroscience, Piazza dell’Ateneo Nuovo 1, 20126, Milano, Italy

**Keywords:** Cognitive maps, Spatial representations, Underground stations, Associative-learning mechanisms, Semantic memory, Distributional semantic models

## Abstract

Recent evidence has indicated that spatial representations, such as large-scale geographical maps, can be retrieved from natural language alone through cognitively plausible distributional-semantic models, which capture word meanings through contextual relationship (i.e., non-spatial associative-learning mechanisms) in large linguistic corpora. Here, we demonstrate that spatial information can be extracted from purely linguistic data even at the medium-scale level (e.g., landmarks within a city). Our results indeed show that different spatial representations (i.e., with information encoded either in terms of relative spatial distances or absolute locations defined by coordinate axes) of the underground maps of five European cities can be retrieved from natural language. Furthermore, by selectively focusing on the London tube, we show that linguistic data align effectively with both geographical and schematic visual maps. These findings contribute to a growing body of research that challenges the traditional view of cognitive maps as primarily relying on specialized spatial computations and highlight the importance of non-spatial associative-learning mechanisms within the linguistic environment in the setting of spatial representations.

## 1 Introduction

When navigating from one place to another, humans often rely on mental representations of the spatial structure of the environment. These internal representations, encompassing an individual’s (typically distorted) knowledge about the absolute locations of landmarks in space and their relative distance, have been commonly defined as “cognitive maps”, a term originally coined by Tolman (Tolman, 1948; see also: O’Keefe and Nadel, 1978). Over the past few decades, evidence has been steadily building to support Tolman’s work on cognitive maps, with a notable acceleration in more recent years. These discoveries have led to the identification of a rather precise neural substrate for such maps, with key roles played by the hippocampus and entorhinal cortex, brain regions populated by function-specific cells that operate through spatial computations enabling flexible mapping of environments and simulations of trajectories (Derdikman and Moser, 2010; Stoewer et al., 2022). Interestingly, recent proposals suggest that the same neurocognitive system supporting spatial navigation would be recruited also to organize and represent non-spatial conceptual knowledge (Bellmund et al., 2018; Bottini and Doeller, 2020). However, against the priority assigned to spatial computations (mainly influenced by research on rodents; e.g., Tolman, 1948; O’Keefe and Dostrovsky, 1971), other accounts have rather pointed to the importance of domain-general, non-spatial associative learning mechanisms in the formation of cognitive maps (Rinaldi and Marelli, 2020). These proposals directly point to how different sources of information and cognitive mechanisms may interact for structuring spatial knowledge (Friedman and Brown, 2000). More broadly, we align with those positions suggesting that human spatial cognition should be considered inherently multimodal, stemming from the integration of different sources of information, which often leads to systematic simplifications and distortions (Arleo and Rondi-Reig, 2007; Tversky, 2003). Nevertheless, while the role of spatial mechanisms in structuring knowledge is relatively well understood, the contribution of other sources of information and mechanisms remain much less clear.

Notably, recent studies exploiting distributional semantic models (DSMs) indicate that perceptual and spatial information can be boot-strapped solely from the statistical structure of natural language (i.e., a non-spatial domain; see Louwerse, 2018 for a review). These works hark back to previous evidence showing that it is possible to construct spatial maps from verbal descriptions (e.g., Ferguson and Hegarty, 1994; Taylor and Tversky, 1992a, 1992b). In DSMs, word meanings are represented as high-dimensional vectors, which can be induced from the distributional history of words in large collections of natural language data, namely linguistic corpora (Jones et al., 2015; Landauer and Dumais, 1997; Lenci, 2018). The theoretical foundation of this approach lies in the distributional hypothesis, according to which similar words will tend to appear in similar linguistic contexts (Harris, 1954). As such, by quantifying a word’s distribution over linguistic contexts it is possible to capture its meaning. In DSMs, high-dimensional numerical vectors representing words will populate a common multidimensional (semantic) space: the distance (i.e., cosine of the angle) between vectors representing words can be considered as a proxy for their semantic similarity. That is, the closer the vectors in the multidimensional space, the higher their similarity in terms of distributional history (i.e., the more they are semantically related; Lenci, 2018). This is empirically supported by data showing that vector distances from DSMs strongly correlate with human semantic similarity ratings (Baroni et al., 2014; Landauer and Dumais, 1997; Pereira et al., 2016); moreover, DSMs can thoroughly predict human performance in a variety of tasks including semantic priming (Lund and Burgess, 1996; Lapesa and Evert, 2013; Gatti et al., 2022a; Günther et al., 2016), false memories (Gatti et al., 2022b) and free association paradigms (Jones et al., 2018). Indeed, recent DSMs models are built on psychologically-plausible, associative learning mechanism (Günther et al., 2019; Mandera et al., 2017; Rinaldi and Marelli, 2020) and can be conceived as computationally-implemented theoretical frameworks of the structure of human semantic memory (Günther et al., 2019; Jones et al., 2015). Importantly, these models infer meanings from statistical patterns of word usage in natural language only, and do not directly access nor compute spatial relationships. As such, any representation retrieved from DSMs is, by definition, not grounded on spatial computations.^1^

Interestingly, various studies indicate that it is possible to successfully reconstruct the spatial layout of geographical maps from text corpora, suggesting in turn that the structure of the physical world (e.g., relative distances between landmarks) can be bootstrapped from the statistical structure of natural language (Avery et al., 2021). For instance, Louwerse and colleagues could reproduce the structure of geographical maps from various world regions using American (Louwerse and Zwaan, 2009), French (Louwerse et al., 2006), Chinese and Arabic (Louwerse et al., 2012) text corpora. Moreover, by applying this method to the Indus script, Recchia and Louwerse (2016) accurately estimated the relative locations of archaeological sites in the Indus Valley. Similar findings were achieved when investigating the geography of the fictional Middle Earth from *J. R. R. Tolkien’s* books (Louwerse and Benesh, 2012).

Over and above these relative spatial distances, it was shown that Euclidean properties of geographical maps (i.e., absolute locations defined by coordinates axes) are also encoded in linguistic data (Gatti et al., 2022c). The distinction between relative and absolute spatial location is cognitively relevant, as people might use different beliefs and strategies when reasoning about various types of spatial estimates (Friedman and Montello, 2006). While relative locations measure proximities without the identification of spatial anchors (e.g., the distance between two cities), absolute locations identify specific points in Euclidean space (e.g., the latitude and longitude coordinates of target cities; Peer et al., 2021). In particular, the method proposed by Gatti et al. (2022c) consists in identifying words that act as spatial anchors (e. g., *North* and *South* for latitude, *East* and *West* for longitude). A new representation is then constructed by computing the relative similarity in language (through DSMs) between elements populating a target domain (e.g., city names) and these spatial anchors. By applying this method, it has been shown that the spatial positions (latitude and longitude coordinates) of European cities can be largely reproduced solely from language, and thus without the need of spatial computations (Gatti et al., 2022c). Moreover, the spatial distortions characterizing these language-based maps were shown to reflect biases in the mental representation of geographical maps, with linguistic information better accounting for participants’ chronometric performance in spatial tasks compared to real geographic information (Gatti et al., 2022c).

Beside this, it should be acknowledged that, in principle, even smaller geographical units – such as the absolute location of landmarks within cities or neighborhoods and their relative distance (Peer et al., 2019) – could be retrieved from language. Indeed, the ability to successfully reconstruct spatial maps from language should not depend on the scale of the space to be mapped, but rather on the extent (i.e., relative occurrence of words describing specific locations) to which spatial information is encoded within language. Interestingly, evidence suggests that different cognitive processes and neural structures may be involved in the representation and processing of spatial information at different scales (Tversky et al., 1999; Zacks et al., 2000; Peer et al., 2019). It is worth noting that, in cognitive research, there are no strict definitions for different spatial scales (but see Kuipers, 1977 for a common conceptualization of small and large spaces). This is likely due to the hierarchical and relative nature of spatial environments. For instance, while rooms and countries are typically termed as small and large spaces respectively, these classifications would shift if one considered even larger contexts (e.g., objects in the solar system). In this study, we adopt the (approximate) definition of medium-scale spaces used by Peer et al. (2019) to refer to cities and neighborhoods, using networks of subway stations as landmarks to structure this space.

Crucially, several factors hinder the feasibility of reconstructing medium-scale spaces from natural language data. First – and of utmost relevance – it appears that large-scale environments are more frequently represented in linguistic corpora as compared to medium ones (e.g., the frequency of word vectors associated with cities is often higher as compared to those of their respective district^2^). This may be due to the fact that the linguistic corpora used to train the models might reflect the aggregate experience of the whole cohort of speakers of a given language. Arguably, discussions pertaining to large urban centers or countries involve a larger number of speakers, resulting in more prevalent representation in the corpora. Conversely, specific neighborhoods may be discussed primarily by residents, leading to comparatively lower representations. Adding to this, spatial knowledge about large geographical spaces could be highly encoded in language because it cannot be learned through direct exploration, while spatial knowledge about medium spaces that can be traveled from a first-person perspective can, in principle, be induced through actually navigating such spaces (Peer et al., 2019). Another concern arises from polysemous words, i.e., words that possess multiple meanings (Ravin and Leacock, 2000). Although polysemy is a common feature of natural language, traditional DSMs assign a single vector to each word, encompassing all its associated meanings over different contexts contained within the linguistic corpora (Arora et al., 2018; Boleda, 2020). In the case of common urban landmarks, particularly underground stations – designated as reference points of the environment in the current study – it is often observed that their name conveys multiple meanings. For instance, the high-dimensional vector attributed to “Angel” embeds not only its usage for the geographical location of the Central London district, but also encompasses the representation of a spiritual being. In this regard, it is worth recognizing that vectors are distributed representations encoding a word’s learning history, thus allowing DSMs to account for different subordinate meanings (Griffiths et al., 2007; Günther et al., 2019; Günther and Marelli, 2022). However, in the specific context of our study, polysemy introduces potentially troublesome noise in the representations, particularly because the information encoded in vectors may not be predominantly about space. For example, while “Elephant” is likely to occur in contexts discussing it as the largest living land animal, its representation occurring in “Elephant & Castle”, referring to a station in south London, is reasonably captured less accurately. Adding to this, it remains challenging for traditional DSMs to produce semantic embeddings for multi-word phrases (Henry et al., 2018; Pagliardini et al., 2018). Crucially, multi-word phrases are not rare in urban landmarks names (e.g., consider again “Elephant & Castle”). In particular, the training process of *fastText*, the DSMs used in the current study, takes into account subword units of character n-grams of length 5 to generate word representations. Thus, when retrieving a multi-word expression like “Elephant & Castle,” *fastText* computes it as a single word and applies the same character n-gram representation approach. That is, the model would compute the vectors for these character n-grams and then combine them to obtain a single vector representation (Bojanowski et al., 2017).

As a result, while the retrieval of spatial layouts of large-scale environments is a reliable process, it seems challenging for traditional DSMs to produce similar patterns for medium-scale environments, as these are i) mapped with a lower frequency in language (as compared to large-scale environments), ii) populated by polysemous words at a very deep level, and iii) include locations with labels comprising more than one word. In light of these challenges, the relevant literature has been focused insofar on large-scale spaces, leaving open the question of whether also medium-scale spaces can be retrieved from natural language. This open question is of crucial importance for the study of spatial cognition. Indeed, medium-scale environments assume a central role in human daily navigation, even more so than larger geographical spaces (e.g., between cities in different countries). Indeed, individuals routinely interact and rely on common urban landmarks such as stations, bus stops and different buildings to navigate and orient themselves in these surroundings (Tom and Denis, 2004).

Consistent with this perspective, research investigating spatial cognition in humans has often focused on navigation abilities assessed in rooms or urban settings (Ekstrom and Isham, 2017; Chrastil, 2013; Peer et al., 2019). Similarly, studies investigating the effects of language on navigation have primarily centered on tasks within small to medium contexts, while exploiting different types of spatial language or task-relevant verbal cues (Tom and Denis, 2004; Hermer-Vazquez et al., 2001; Pyers et al., 2010; Shusterman et al., 2011). However, linguistic information can influence spatial cognition through multiple mechanisms, with the relevance of such mechanisms that may vary as a function of the spatial scale under investigation (e.g., see: Bellmund et al., 2018; Ekstrom and Isham, 2017). In essence, there are discernible differences in the focus of research on linguistic information shaping spatial representations. Whereas some studies have delved into the use of spatial language in small to medium scale environments, others have explored domain-general, non-spatial associative learning mechanisms inherent in language for the development of large-scale maps representation (i.e., by means of DSMs). In light of the pivotal role of medium-scale spaces in the study of spatial cognition, it is crucial to fill this gap by extending the exploration of such domain-general mechanisms at the medium-scale level. This exploration directly contributes to our understanding of how maps are built and shaped through experience.

Importantly, spatial knowledge can be acquired not only through direct navigation but also from cartographic maps, namely, visual representations of an environment (Kraak and Fabrikant, 2017). Evidence has been provided suggesting a superiority of learning from cartographic maps, as compared to direct exploration, for judgments about relative location and straight-line distances (Thorndyke and Hayes-Roth, 1982) as well as for the development of mental representation of one’s city of residence (Frankenstein et al., 2012; cf.: Byrne et al., 2007). In contrast to direct navigation, which relies on multiple direct sensory experiences, cartographic maps are mainly perceived visually. However, most city maps typically provide minimal visual details of locations (Frankenstein et al., 2012), with the overarching aim of reducing complexity to facilitate user comprehension and journey planning (Roberts et al., 2016). This simplification is especially evident when it comes to transport networks maps, where schematic representations prevail. For example, lines are generally portrayed as straight-lines and sharply radiused corners. As a result, it is commonly assumed that the representation of underground stations on schematic maps (particularly studied in the context of the London underground) deviates from their true geographical locations (Jenny, 2006; Guo, 2011; Longo, 2022). Of notable concern are the findings reported by Guo (2011): through the manual measurement of map distances for each station-to-station link, the author reported a low correlation coefficient (e.g., of only .22) between map and geographical distances within the London underground; an observation implying substantial distortions in the visual representation of spatial relationships on the map.

Crucially, linguistic processes are often coupled with visual input, as evident in everyday activities such as engaging in conversations or following directions on a map while navigating through a city (Coco and Keller, 2009). However, evidence to date is missing concerning whether the distributional history of words can also capture visual information of schematic, cartographic maps; and, if so, whether language-derived spatial representation differently reflect geographical or schematic map distances. This question is of utmost interest in the specific context of our study in light of prior evidence showing that spatial information within language provides distinct contributions in the organization and incorporation of biases in human spatial representations (Gatti et al., 2022c; Gatti et al., 2024). Finally, the actual discernibility of schematic map distortions may vary when considering the distance between stations or their absolute positions: a possibility that has not been thoroughly addressed insofar in the literature.

The present study aims to test these unresolved issues. We thus explored whether spatial information can be extracted from linguistic data at medium-scale level, across two simulation studies: in Experiment 1, we explored whether DSMs could reproduce the spatial structure (namely, distances between stations) of the undergrounds of five European cities; in Experiment 2, we investigated whether DSMs can reproduce the Euclidean properties (namely, latitude and longitude coordinates of stations) of the same five European undergrounds. Then, in Experiment 3, we aimed to (i) replicate prior evidence suggesting relevant distortions between geographical and map distances between stations in the London underground, as well as investigating whether linguistic data differently capture geographical or schematic map visual information (Experiment 3A); (ii) assessing whether geographical vs. schematic map distortions can be discerned also at the absolute position level (Experiment 3B).

## 2 Experiment 1

In Experiment 1, we explored whether DSMs could reproduce the spatial structure of medium-scale geographical maps. In particular, we focused on the undergrounds of five European cities: Berlin, London, Madrid, Milan, and Paris. For each city, and for each pair of stations, we considered both the real geographical and the linguistic distance. The latter were retrieved from DSMs applied to 5 different languages (German, English, Spanish, Italian, and French) through *fastText* (Bojanowski et al., 2017) in its 2018 version (Grave et al., 2018), a distributional-semantic model for which word vectors in several languages were made available (see below for further details).

### 2.1 Methods

#### 2.1.1 Stimuli

Stimuli included the names of underground stations from five European cities: Berlin, London, Madrid, Milan and Paris. Since the DSM we used was implemented in 2018, we only considered those stations being active before that year (including 2018).

##### Berlin

One hundred and seventy-five Berlin underground stations were included. Station names were retrieved from BerlinOpenData (https://daten.berlin.de).

##### London

Two hundred and sixty-five London underground stations were selected as stimuli. The name of each station was retrieved from OpenStreetMap (https://wiki.openstreetmap.org/wiki/List_of_London_Underground_stations). From the selected database, stations from the overground railway systems (i.e., London overground, east London and Thameslink) as well as duplicated records were excluded (i.e., stations for which coordinates of both the entrance and the platform were reported; in such cases, the platform coordinates were used).

##### Madrid

Two hundred and forty-one Madrid underground stations were included. Station names were retrieved from Madrid Open-MobilityData (https://transitfeeds.com/).

##### Milan

One hundred and seven Milan underground stations were included. Station names were retrieved from Milan municipality open-data website (https://dati.comune.milano.it/)

##### Paris

Two hundred and ninety-five Paris underground stations were included. Station names were retrieved using the *paris_metro* R function from the *ggmaptile* R package (Taylor, 2022).

Geographical positions (i.e., latitude and longitude coordinates) and names of each underground station were used to compute geographical and linguistic distances, respectively.

#### 2.1.2 Computation of real geographical distances

Among each set of underground stations, the selected stations were paired one to another for a total of 15225 pairs for Berlin, 34980 pairs for London, 28929 pairs for Madrid, 5671 pairs for Milan, and 43365 pairs for Paris. Real geographical distances for each station pair were computed using the *raster* R package (Hijmans, 2022), which estimates distances in kilometers starting from longitude and latitude coordinates.

#### 2.1.3 Distributional-semantic model

The distributional-semantic model used here was *fastText* (Bojanowski et al., 2017; Grave et al., 2018). The model was trained on Common Crawl (around 630 billion words) and Wikipedia (around 9 billion words) using the Continuous Bag of Words (CBOW) method, an approach originally proposed by Mikolov and colleagues (2013), with position-weights across 300 dimensions, with character n-grams of length 5 and a window size of 5. When using CBOW, the obtained vector dimensions capture the extent to which a target word is reliably predicted by the contexts in which it appears. With respect to traditional distributional models, whose ability to generate high-quality distributed semantic representations is limited to words that are sufficiently frequent in the input data, *fastText* is based on the idea (originally proposed by Schutze, 1993; and realized by Bojanowski et al., 2017) to take into account sub-word information by computing word vectors as the sum of the semantic vectors for the n-grams associated with each word. For each city, we retrieved the pre-trained vector for each station (first letter capitalized) in its associated language (i.e., German for Berlin, English for London, etc.) from the most recent multilanguage *fastText* semantic spaces available (Grave et al., 2018). Since *fastText* is able to differentiate between uppercase and lowercase letters, we kept the main names with the first letter capitalized, conjunctions or prepositions in lowercase font, and the space maintained in multi-word labels (e.g., Bromley by Bow).

#### 2.1.4 Computation of linguistic distances

To compute linguistic distances, we first retrieved from *fastText* the vector associated to each station. Then, for each stations pair the linguistic distance was computed as the cosine similarity between the vectors of either element of the pair subtracted from 1 (i.e., hence transforming proximity into distance: the lower the value, the closer the two vectors as predicted by the model). Vectors were retrieved using the *fastTextR* R package (Schwendinger and Hvitfeldt, 2022), while linguistic distances were computed using the *dist* function of the *proxy* R package (Meyer and Buchta, 2021).

### 2.2 Data analysis and results

All the analyses were performed with RStudio (RStudio Team, 2015). Using the *lme4* R package (Bates et al., 2015), we estimated a linear mixed model having geographical distance as the dependent variable, linguistic distance as continuous predictor, and city as categorical predictor along with their interaction. The two stations comprised in the pair were set as random intercepts. Specifically, in the *lme4* syntax the model estimated was:

$$\begin{array}{c} \;Geographical\_distance\;\sim \;Linguistic\_distance\;\times \;City\;+(1|station1)\\ +(1|station2) \end{array}$$

Results showed a main effect of linguistic distance, *F*(1,128133) = 124.70, *p* < .001, and city, *F*(4,3888) =112.30, *p* < .001. The interaction between linguistic distance and city was also significant *F* (1,127885) = 12.50, *p* < .001, indicating that the effect of linguistic distance on geographical distance differed between the cities tested.

**Fig. 1:**
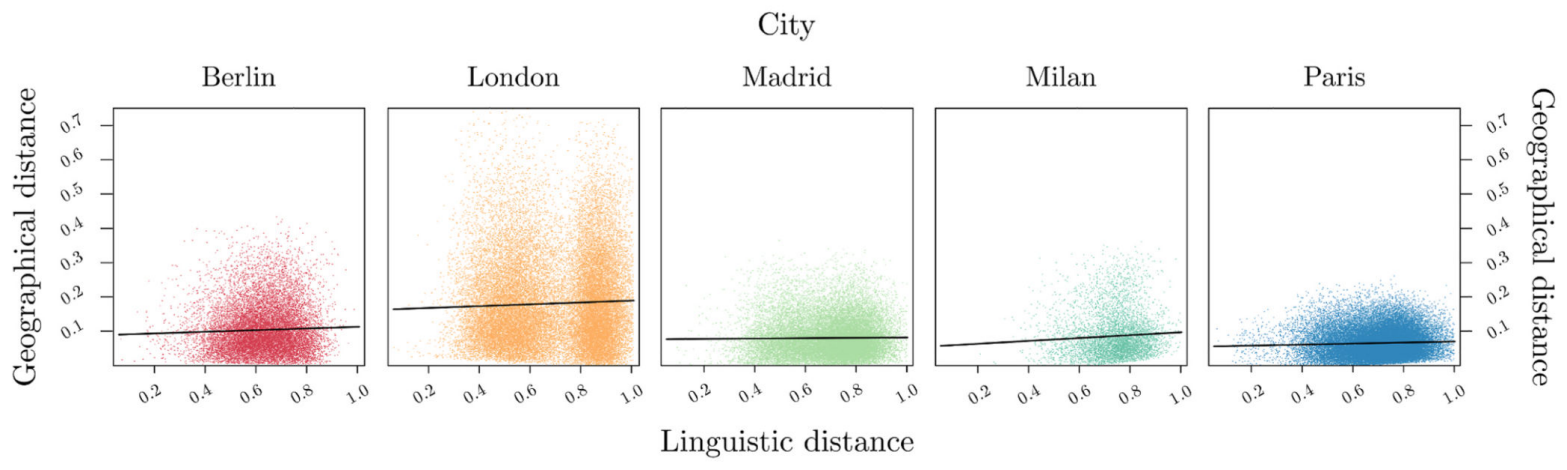
Plot illustrating the positive relationship between geographical and linguistic distances for the underground stations of the five cities tested. Each dot represents a specific pair of stations. Note that the visual appearance of slopes should be interpreted in light of post-hoc results.

Post-hoc analyses revealed a significant positive relationship between linguistic distance and geographical distance for Berlin, *b* =.024, *SE* = .004, *z* = 5.84, *p* < .001, London, *b* = .027, *SE* = .002, *z* = 15.31, *p* < .001, Milan, *b* = .042, *SE* = .008, *z* = 5.20, *p* < .001, and Paris, *b* = .015, *SE* = .003, *z* = 5.52, *p* < .001, indicating that higher linguistic distances correspond to higher geographical distances. By contrast, no significant relationship emerged between linguistic distance and geographical distance for Madrid, albeit the direction was compatible with the expected pattern, *b* = .005, *SE* = .003, *z* = 1.61, *p* = .10. These results suggest that the structural organization of underground stations, embedding the information about the distance between two stations, can be retrieved from language, therefore extending previous findings from the literature focused on the distance between cities (Avery et al., 2021; Louwerse and Zwaan, 2009) at a medium-scale level.^3^ However, this relationship between linguistic and geographical distance varies depending on the specific city assessed. Fig. 1 shows the relationship between linguistic and geographical distance for the underground stations of the five cities tested.

## 3 Experiment 2

In Experiment 2 we explored whether DSMs could reproduce the Euclidean properties of medium-scale geographical maps. In particular, the same 5 European undergrounds of Experiment 1 were tested, extracting for each station both the real geographical coordinates (i.e., latitude and longitude) and the language-based coordinates.

### 3.1 Methods

#### 3.1.1 Stimuli

Stimuli were identical to those used in Experiment 1. The real geographical coordinates of each station were then matched with the language-based coordinates (i.e., linguistic latitude and longitude; see below for details on the computation of these coordinates) extracted from *fastText*.

#### 3.1.2 Distributional-semantic model

As in Experiment 1, the distributional-semantic model used was *fastText* (Bojanowski et al., 2017). In addition, we extracted the vector representations for words describing the four cardinal points, namely, *North, South, East*, and *West* (different across English, German, Spanish, Italian and French; for further details see: https://osf.io/3hvz7/). For each language, cardinal points names were translated from English using NorthEuraLex (i.e., a multilanguage database, Dellert et al., 2019).

#### 3.1.3 Computation of language-based coordinates

For each station, we computed a linguistic latitude and a linguistic longitude value (as in Gatti et al., 2022). Linguistic latitude of a station *k* was obtained with the following formula:

$$cos(\vec{k},\vec{\;North\;})-cos(\vec{k},\vec{\;South\;})$$

Thus, we subtracted from the cosine (cos) of the angle formed by the vectors representing a given station $\left(\vec{k}\right)$and the word *North*, the cosine of the angle formed by the vectors representing the same station $\left(\vec{k}\right)$ and the word *South*. Positive values thus indicate a northern position according to language usage (i.e., mimicking geographical latitude).

Similarly, linguistic longitude was obtained with the following formula:

$$\text{cos}(\vec{k},\vec{\;East\;})-\text{cos}(\vec{k},\vec{\;West\;})$$

Also in this case, positive values will thus indicate an eastern position according to language usage (i.e., mimicking geographical longitude).

### 3.2 Data analysis and results

All the analyses were performed using RStudio (RStudio Team, 2015). We estimated two linear models (i.e., one for each spatial coordinate) having city-level z-transformed geographical latitude or geographical longitude^4^ as the dependent variable, linguistic latitude or linguistic longitude as continuous predictor, and city as categorical predictor, as well as their interactions. Specifically, in the *lme4* syntax the models estimated were:

$$\begin{array}{l} \;Z.Geographical\_Latitude\;\sim \;Lingustic\_Latitude\;\times \;City\;\\ \;Z.Geographical\_Longitude\;\sim \;Linguistic\_Longitude\;\times \;City\; \end{array}$$

The main effect of linguistic latitude on geographical latitude was significant, *F*(1,1073) = 7.44, *p* = .006. indicating that higher linguistic-latitude values (i.e., linguistically northern locations) correspond to higher geographical-latitude values (i.e., geographically northern locations). No significant main effect of city, *F*(4,1073) = .24, *p* = .91, and no significant linguistic latitude by city interaction, *F*(4,1073) = 1.50, *p* = .19, were found.

Similarly, the effect of linguistic longitude on geographical longitude was significant, *F*(1,1073) = 3.90, *p* = .048, indicating that higher linguistic-longitude values (i.e., linguistically eastern locations) correspond to higher geographical-longitude values (i.e., geographically eastern locations). No significant main effect of city, *F*(4,1073) = .39, *p* = .81, and no significant linguistic longitude by city interaction, *F* = (4,1073) = 2.10, *p* = .079 were found.

**Fig. 2:**
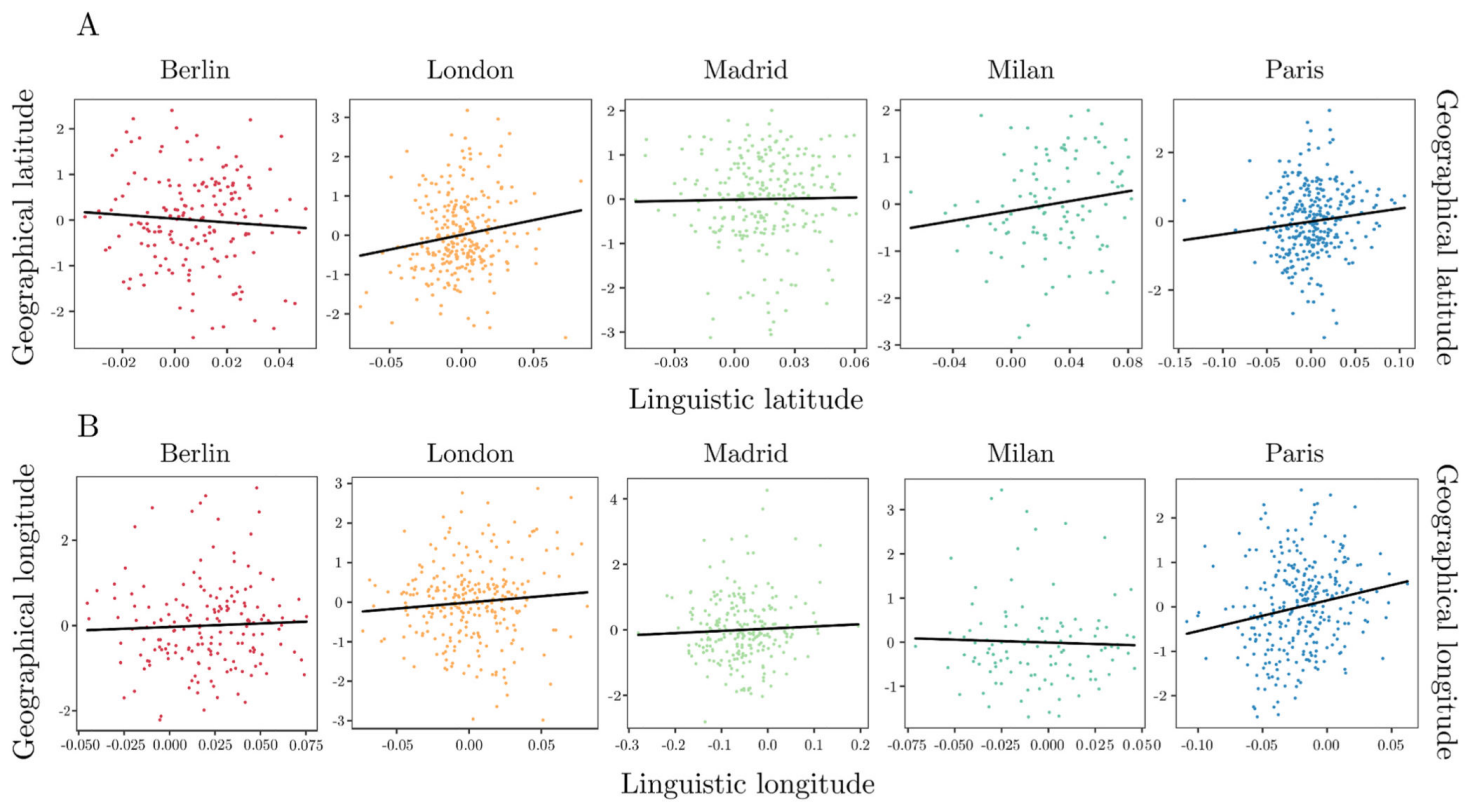
Plot illustrating the positive relationship between (a) geographical and linguistic latitude, and (b) geographical and linguistic longitude for the stations of the five cities included. Each dot represents a specific subway station. Note that any apparent difference in slopes does not reach significance at the interaction level.

Together, these findings suggest that Euclidean properties (latitude and longitude coordinates of stations) of geographical medium-scale map are encoded in linguistic data, thus extending previous evidence on large-scale environments (Gatti et al., 2022). Fig. 2A and B shows the relationship between A) geographical and linguistic latitude, and B) geographical and linguistic longitude for the stations and the cities tested.

## 4 Experiment 3

In Experiment 1 and Experiment 2 we showed that different properties (distances and absolute positions of landmarks) of spatial representations of medium-scale spaces can be retrieved from natural language. However, an open question remains about whether information from visual representations of these spaces (i.e., cartographic maps) might be better reflected in language. This issue gains particular significance given prior evidence suggesting distortions between the representation of underground stations on maps and their actual locations (Guo, 2011; Longo, 2022). Nevertheless, the discernibility of these distortions may vary when considering the distances between stations or their absolute positions. Thus, in Experiment 3 we aimed to (i) replicate prior evidence suggesting relevant distortion between geographical and schematic map distances between stations in the London underground, as well as investigating whether linguistic data differently capture geographical or map information (Experiment 3A); (ii) assessing the actual discernibility of potential geographical vs. schematic map distortions of the London underground when the absolute positions of stations are considered (Experiment 3B).

### 4.1 Experiment 3A

In Experiment 3A we aimed to replicate prior evidence suggesting relevant distortion between geographical and schematic map distances between stations in the London underground, as well as investigating whether linguistic data differently capture geographical or map information.

### 4.2 Methods

#### 4.2.1 Stimuli

The same two hundred and sixty-five London underground stations of the previous experiments were selected as stimuli. The previously computed geographical and linguistic distances were matched with the cartographic map-based distances between each station pair (see below for details on the computation of these distances).

#### 4.2.2 Computation of schematic map-based distances

For the computation of schematic map-based distances, we first computed coordinates of stations from the official map of the London underground (https://tfl.gov.uk/maps/track/tube) by exploiting a web-based tool which allows the extraction of graphical data (e.g., X and Y coordinates) from plots, images, and maps (https://automeris.io/WebPlotDigitizer/). Then, schematic map-based distances for each station pair were computed using the *raster* R package (Hijmans, 2022), which estimates distances in kilometers starting from longitude and latitude coordinates (e.g., in this case, X and Y coordinates).

### 4.3 Data analysis and results

All the analyses were performed using RStudio (RStudio Team, 2015). First, we investigated the relationship between geographical distance and map-based distance by estimating a linear mixed model having schematic map-based distance as dependent variable and geographical distance as continuous predictor. The two stations comprising the pair were set as random intercepts. Specifically, in the *lme4* syntax the model estimated was:

Map_distance~Geographical_distance+(1|station1)+(1∣station2)

**Fig. 3:**
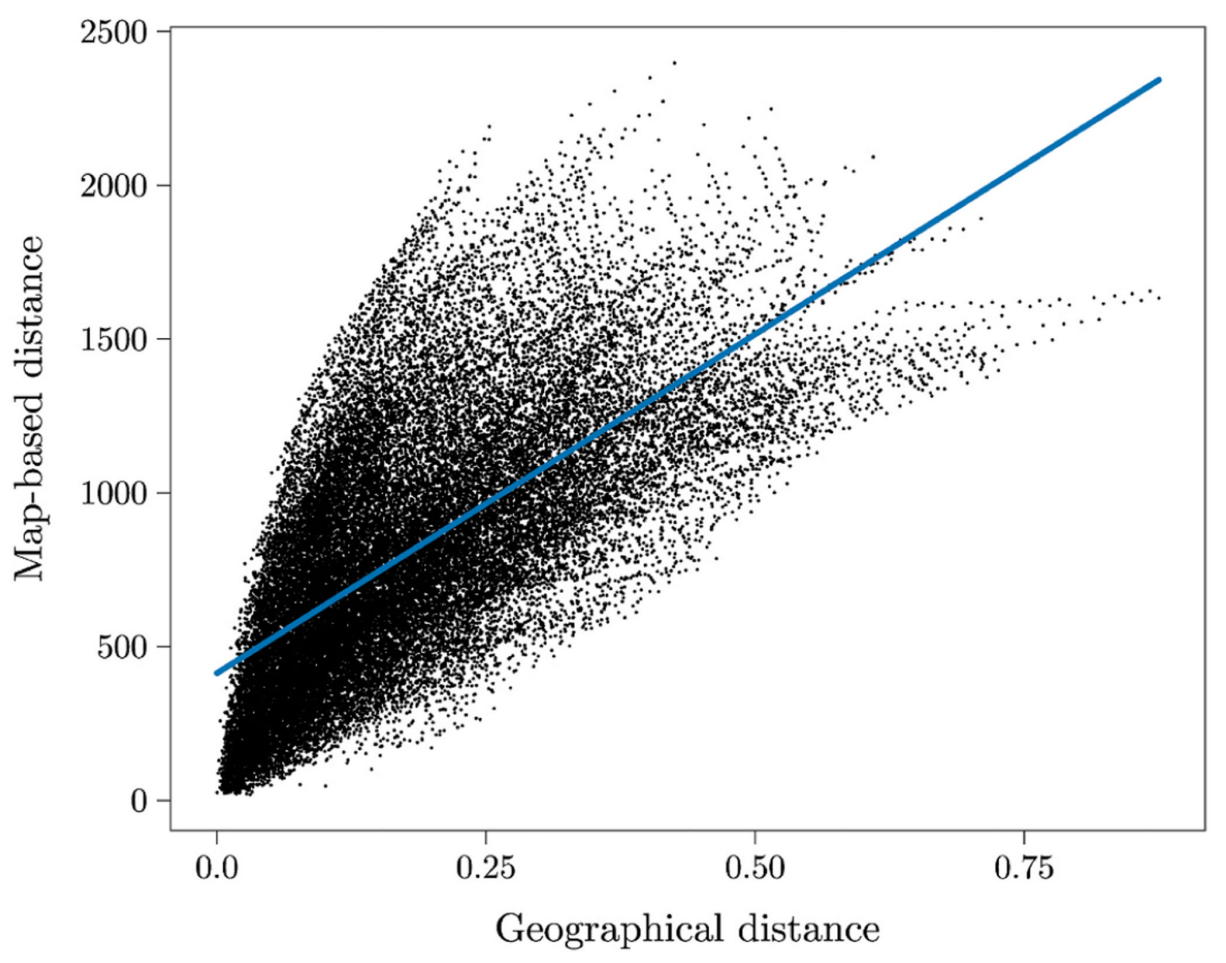
Scatterplots of the positive relationship between geographical distance and schematic map-based distance. Each dot represents a specific pair of the London underground stations.

Results showed a positive relationship between map distance and geographical distance, *β* = .89, *b* = 2936.27, *SE* = 15.94, *t*(34643.75) = 184.21, *p* < .001, (Fig. 3). Thus, contrary to previous evidence (Guo, 2011; see also Longo, 2022) documenting a weak relationship, our findings suggest a close correspondence between geographical and map-based distances.

Then, we estimated a linear mixed model having z-transformed distances^5^ as the dependent variable, linguistic distance as continuous predictor, and type (i.e., geographical and schematic map-based distances) as categorical predictor, as well as their interaction. The two stations comprising the pair were set as random intercepts. Specifically, in the *lme4* syntax the model estimated was:

Z.Distance~Lingustic_distance×Type+(1∣station1)+(1∣station2)

**Fig. 4:**
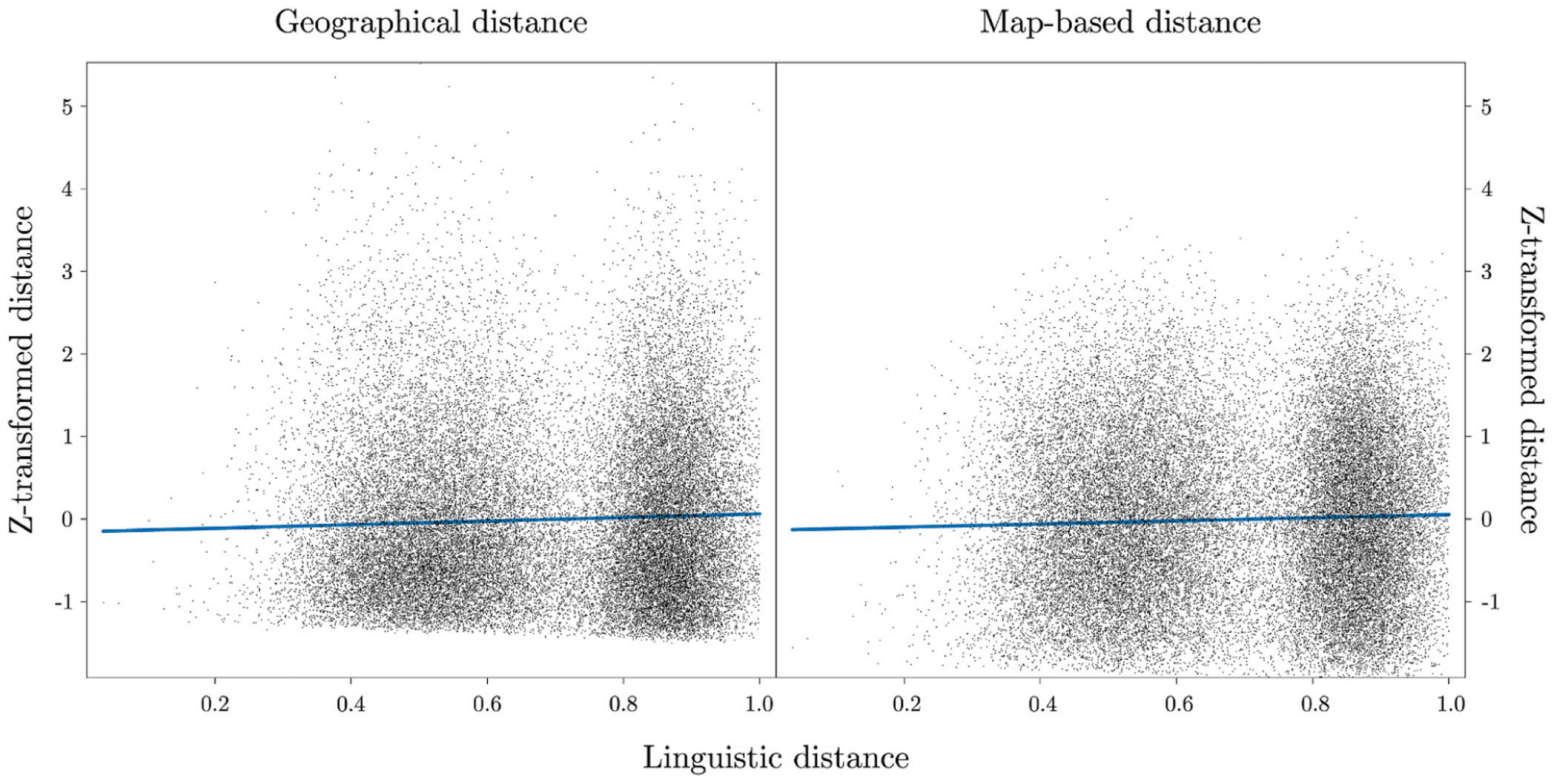
Plot illustrating the positive relationship between (left panel) geographical and linguistic distance, and (right panel) schematic map-based and linguistic distance. Each dot represents a specific pair of the London underground stations.

The main effect of linguistic distance on z-transformed distance was significant, *F*(1, 69906) = 115.39, *p* < .001. indicating that higher linguistic distances values correspond to higher distances values. On the other hand, the main effect of type, *F*(1, 69428) = .769, *p* = .380, as well as the linguistic distance by type interaction, *F*(1, 69428) = .828, *p* = .178, were not significant (Fig. 4). These findings suggest that actual geographical and schematic map-based distances are similarly encoded within the linguistic data.

### 4.4 Experiment 3B

Building upon the insights gained from Experiment 3A, we conducted a second experiment, this time focusing on absolute positions.

### 4.5 Methods

#### 4.5.1 Stimuli

The same two hundred and sixty-five London underground stations of the previous experiments were selected as stimuli. The previously computed geographical and linguistic coordinates were matched with the map-based coordinates between each station pair (see below for details on the computation of these coordinates).

#### 4.5.2 Computation of schematic map-based coordinates

**Fig. 5:**
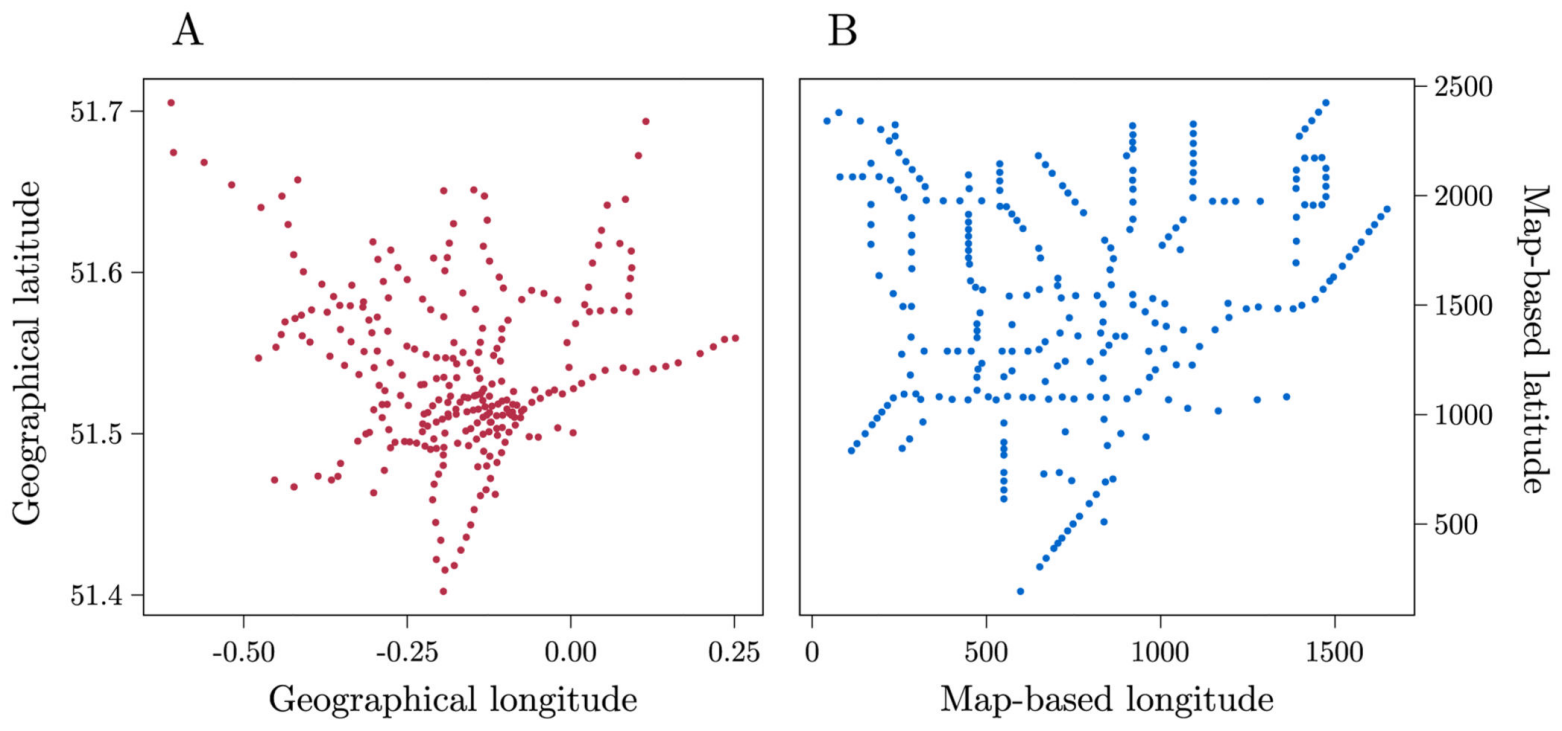
Plot illustrating the geographical (A) and schematic map-based coordinates (B) for each station of the London underground.

Map-based coordinates were computed as described in Experiment 3A. For geographical and schematic map-based coordinates, see Fig. 5A and B, respectively.

### 4.6 Data analysis and results

**Fig. 6:**
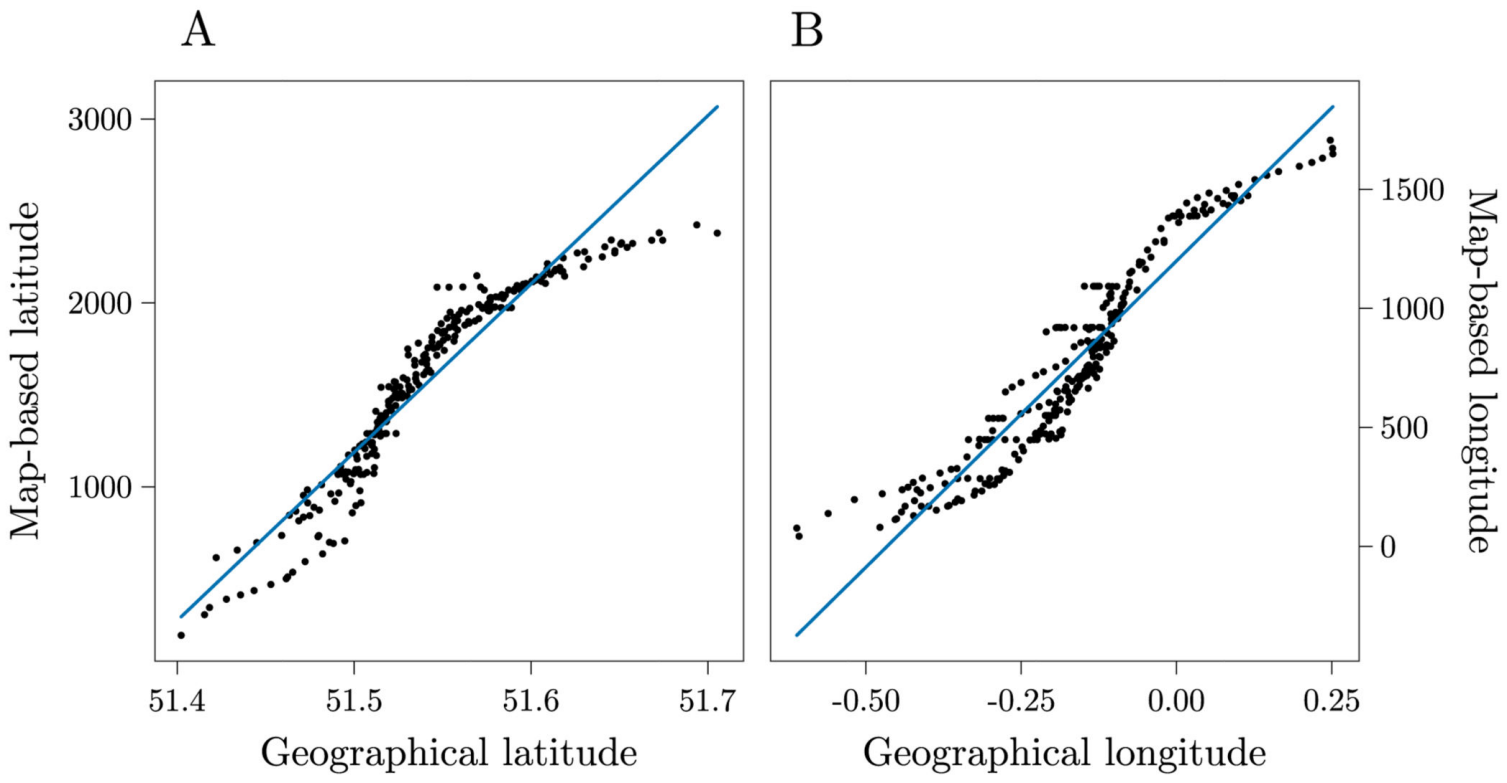
Scatterplots of the correlations between geographical and schematic map-based latitude (a), and between geographical and schematic map-based longitude (b). Each dot represents a specific station of the London underground system.

All the analyses were performed using RStudio (RStudio Team, 2015). To investigate the relationship between the schematic map-based coordinates and the geographical coordinates we computed Pearson correlation coefficients. Our results showed a high correlation between the map coordinates and the geographic coordinates (all *p*s < .001). Specifically, the correlation between schematic map-based latitude and geographical latitude had *r* = .95, while the correlation between schematic map-based longitude and geographical longitude had *r* = .95 (Fig. 6A and B, respectively). This indicates that the London underground’s official map faithfully represents the geographical coordinates of stations, suggesting in turn that any potential distortion in the map is not discernible at the absolute positions level.

## 5 Discussion

Recent studies have demonstrated that the construction of cognitive maps may not predominantly build on specialized spatial computations, thus challenging the theoretical accounts assigning priority to spatial mechanisms (Avery et al., 2021; Gatti et al., 2022c). In line with this perspective, different works leveraging on DSMs, emphasized the importance of non-spatial learning mechanisms that are at play in language experience as pivotal to the formation of cognitive maps (Rinaldi and Marelli, 2020). In fact, linguistic data itself can produce spatial information and can be successfully employed to reconstruct geographical maps, both in terms of their structural (Louwerse, 2018) and Euclidean properties (Gatti et al., 2022c). From a broader perspective, this evidence aligns with those theoretical frameworks suggesting flexible interactions between different sources of knowledge in the development of cognitive representations, including cognitive maps (Louwerse, 2018; Kemmerer, 2015; Clark and Paivio, 1987; Davis and Yee, 2021). Indeed, spatial and linguistic information seem to mutually influence each other in a bidirectional way. For instance, the seminal study by Casasanto (2008) demonstrated that the spatial arrangement of words (e.g., their physical distance) influences humans’ judgments of semantic similarity between the concepts those words represent. On a similar vein, Winter and Matlock (2013) showed that describing fictional cities as similar in political decision made people assume that they were spatially close to each other. Importantly, these results support the idea of a multimodal representation of space, which emerges from vision, sensorimotor interaction, and language, among other sources.

However, evidence assessing the role of linguistic experience (as captured by DSMs) in developing spatial representations has, so far, focused on large-scale spaces, leaving open the question of to what extent medium-scale environments can be retrieved from language. Here, we demonstrated that spatial information can be extracted from purely linguistic data even in the case of medium-scale spaces. Our results indeed showed that through psychologically plausible distributional-semantic models it is possible to capture the structural organization of the underground maps of different European cities, embedding the information about the distance between stations. These findings are in line with previous evidence suggesting that statistical regularities encoded in natural language reflect the structure of the perceptual world we live in (Louwerse, 2011). Crucially, language does not merely encode spatial information but actively contributes to structuring spatial representations. Indeed, by extending prior works in large-scale spaces, our results provide the first evidence that also non-spatial associative learning mechanisms, rather than spatial computation alone, may in principle support the formation of cognitive maps of smaller environments.

This work contributes to empirical efforts and debates attempting to unravel similarities and differences between neurocognitive systems operating across different spatial scales. Competing theories range from proposals of a unified spatial processing system (Hirtle and Jonides, 1985; Worden, 1992) to accounts suggesting distinct mechanisms for different spatial scales (Montello, 1993; Tversky, 2003; but see Peer et al., 2019 for reconciliatory evidence). Additionally, our results relate to proposals challenging a strict isomorphism between patterns of neural activity (e.g., grid cells), cognitive representation, and behaviors (e.g., navigation). While neural activity in structures traditionally associated with spatial navigation is often assumed to reflect a spatial metric representation that directly maps onto cognitive processes and behaviors, this assumption has been questioned (Warren, 2019; Ekstrom et al., 2020). More broadly, these components pertain to different levels of analysis (Marr, 1982), and even precise characterizations at one level do not imply direct correspondence with the others. In our context, while function-specific cells may engage in spatial computations relevant to navigation (Derdikman and Moser, 2010; Stoewer et al., 2022), this does not imply that such neural activity equate how we represent or navigate space. This reasoning aligns with the mereological fallacy (Bennett and Hacker, 2003; Krakauer et al., 2017), which cautions against assuming that properties of specific neural circuits directly translate to whole behaviors. Indeed, rather than primarily relying on spatial computations, complex abilities such as navigating our environment or recalling locations of cities on a map most likely emerge from the flexible interaction of multiple information and processes.

In addition to this, we also showed for the first time that both structural and Euclidean properties of medium-scale maps (i.e., relative spatial distances, and absolute locations defined by coordinate axes, respectively) can be retrieved from natural language. Indeed, our results not only showed that distance-like information between underground stations can be extracted through DSMs, but also that language-based coordinates (i.e., linguistic latitude and longitude) correspond to real geographical coordinates. This supports recent theoretical views suggesting a co-existence of distinct representational structures for encoding spatial information (Peer et al., 2021). Namely, that spatial knowledge could flexibly take the form of environmental elements encoded in Euclidean or structural space.

However, it must be acknowledged that the spatial representations derived from linguistic data for medium-scale spaces are less accurate than those of large-scale geographical maps (e.g., see Gatti et al., 2022c), likely owing to the higher levels of noise inherent in the linguistic data about medium-scale spaces. For direct evidence supporting this see Additional analyses 3A and 3B in the Supplementary Materials, where we demonstrated that DSMs better capture large-scale geographical maps as compared to medium-scale spaces. Because the distributional-semantic models used in the present study rely on distributional patterns of words from natural language, this lower accuracy (in terms of variance explained) is likely determined by the fact that large written corpora (and especially in Web corpora not specifically based on geographical knowledge, as the ones employed here) tend to focus more on larger geographical environments compared to medium (and small) ones. Furthermore, the reconstruction of such medium-scale environments through DSMs is particularly challenging, given the high prevalence of both polysemous and multi-word labels in underground station names. Accordingly, the variability observed between cities in Experiment 1 (i.e., the ability to retrieve distance-like information from language differed across the cities tested) may depend on these factors. Together, our results provide support to the notion that maps can be retrieved from text regardless of the scale to be mapped, despite the availability of specific spatial information in language would likely determine the feasibility of reconstructing such maps. It follows that even smaller environment (e.g., room) can in principle be reconstructed from language, contingent upon the adequateness of the linguistic source, which can depend on various factors, including corpus size and quality, the complexity of the spatial concept, and the richness of the language used; a possibility that needs to be probed by future research. In this regard, an important consideration arises regarding the very type of linguistic source used to capture cognitive maps, particularly in relation to its corpus size. Indeed, while on the one hand these models are trained on huge corpora (way bigger than the amount of words that a speaker would ever encounter), on the other hand this overexposure can be framed as indexing the experience of the whole cohort of speakers of a given language. At the same time, this does not affect the plausibility of the model from an algorithmic point of view (i.e., the algorithm behind model learning approximates human learning). That is, the psychological plausibility of DSMs should be evaluated in light of their ability to capture essential aspects of language learning and representation through associative-learning mechanisms.

Importantly, in everyday life, linguistic and visual processes are frequently intertwined, as evident in common behaviors such as following directions on a city map (Coco and Keller, 2009). Moreover, and of particular interest in the context of our study, it has been consistently reported that transport networks maps, being schematic in nature, introduce significant distortions in the visual representation of stations as compared to their actual geographical locations (Guo, 2011; Longo, 2022). This leads us to address other pivotal questions: namely, can the distributional history of words also capture the visual information inherent in schematic, cartographic maps? If so, does the language-derived spatial representation distinctly reflect geographical or schematic map distances?

Thus, in our investigation, we first aimed to replicate prior evidence highlighting such distortions. Notably, contrary to prior evidence (Guo, 2011; Longo, 2022), our results demonstrate a close correspondence between geographical distances of stations of the London underground and those portrayed in the relative visual schematic map. Furthermore, we observed an even more robust alignment between geographical and schematic map-based coordinates (namely, latitude and longitude for geographical coordinates; Y and X coordinates for schematic maps). These discrepancies with prior literature may emerge from various factors. First, there are considerable variations in the schematic map used in prior studies, which ranges from the iconic one designed by Harry Beck in 1933, to the most recent version available, which undergoes constant updates with additions or modifications to stations and paths. Second, the measurement techniques employed in previous studies are also highly diverse. For instance, the example provided by Longo (2022) applied a D’Arcy Thompson transformation grid (Thomson, 1917) to the iconic 1933 schematic map. On the other hand, Guo (2011) manually measured the distance of each station-to-station link in Adobe Photoshop on a more modern map. Lastly, Jenny (2006) also used their modern-day underground map, but the measurements were carried out with a specific software to construct distortion grids (Beineke, 2001). Interestingly, by applying this methodology, Jenny (2006) found only a partial confirmation of distortions between the geographical and schematic map, thus aligning more with our findings. Finally, our study suggests that natural language similarly encodes spatial information pertaining to the actual geographical and visual schematic representations of the London underground. However, given the substantial alignment observed between the geographical and schematic maps, especially at the absolute position level, the lack of differences may stem from this close correspondence. More generally, schematic maps of underground networks, even if not perfectly, reflect by necessity the actual layout of the respective geographical maps. Hence, our results should be carefully interpreted in light of this consideration.

In conclusion, our study provides evidence that spatial information can be extracted from natural language data alone even for medium-scale environments. Our findings suggest that the ability to successfully reconstruct spatial maps from language is determined by the degree to which spatial information is encoded within language, rather than the spatial scale to be mapped. This insight remains to be tested in small-scale spaces provided a distributional-semantic model trained on sufficiently accurate natural language data, that is, text corpora conveying relevant spatial information at the small-scale level. As a final note, in light of prior evidence highlighting a substantial alignment between systematic biases in humans’ mental representation of large-scale spaces and the distortions encoded natural language (Gatti et al., 2022c), future behavioral studies should explore whether a similar pattern emerges also for mental representation of medium-scale environments. Overall, these findings contribute to a growing body of research that challenges the traditional view of cognitive maps as primarily relying on specialized spatial computations and highlights the significance of non-spatial associative learning mechanisms within the linguistic environment in construction of cognitive maps.

## Supplementary Material

Appendix

## Data Availability

All data, scripts, codes, and materials used in the analysis are available online (https://osf.io/a9wu5/). The preprint version of this article is available online (https://osf.io/preprints/psyarxiv/wdbxs).

## Footnotes

1 Note that DSMs are usually trained on large corpora (e.g., the entire Wikipedia) that incorporate disparate forms of knowledge and descriptions, which also contain explicit spatial references (e.g., London is 958 km from Milan). However, the efficacy of DSMs in capturing concept distances does not hinge on the direct memorization of factual statements, but rather on the mathematical encoding of a word’s distribution over linguistic contexts. This mechanism allows to capture semantic associations encompassing spatial relationships.

2 An example about these frequency ranks from the DSM used in the current study (i.e., *fastText*) is provided in the Supplementary Materials.

3 Note that geographical distances between station pairs were computed as Euclidean distance (i.e., the straight-line distance between two stations). However, considering the topological distance (i.e., the path taken by the subway line, which may include curves and turns between the two stations) may have led to different results. We thus tested this possibility. Results showed that linguistic distances similarly capture Euclidean and topological distances. These analyses can be found in the Supplementary Materials. Yet, because topological distances result in fewer possible station pairs compared to Euclidean distances, we focused on the latter in our analyses to test a space with higher granularity.

4 That is, since the cities were far from each other with respect to latitude and longitude, the transformation used allows to have uniformly distributed dependent variables.

5 That is, since distance type pertains to different scales, the transformation used allows to have uniformly distributed dependent variables.
