# Supplementary material for: A map of words: Retrieving the spatial layout of medium-scale geographical maps through distributional semantics
Source: Neuropsychologia. Author manuscript; Available in PMC 2026 Apr 24. (PMC7619030; doi:10.1016/j.neuropsychologia.2025.109190)
Supplement: Appendix [file EMS213371-supplement-Appendix.pdf]

Appendix A. Supplementary data

Supplementary data to this article can be found online at https://doi.org/10.1016/j.neuropsychologia.2025.109190.
